# Validation of quantitative perfusion cardiovascular magnetic resonance employing deconvolution techniques with Tofts, modified-Tofts, and Fermi function models against ^15^O-water positron emission tomography

**DOI:** 10.1016/j.jocmr.2025.102678

**Published:** 2025-12-24

**Authors:** Masafumi Takafuji, Masaki Ishida, Yasutaka Ichikawa, Satoshi Nakamura, Haruno Ito, Takanori Kokawa, Suguru Araki, Shintaro Yamaguchi, Naoki Hashimoto, Shiro Nakamori, Tairo Kurita, Kaoru Dohi, Hajime Sakuma

**Affiliations:** a Department of Radiology and Mie University Hospital, Tsu, Mie, Japan; b Department of Cardiology and Nephrology, Mie University Hospital, Tsu, Mie, Japan

**Keywords:** Coronary artery disease, Coronary magnetic resonance angiography, Deep learning, Convolutional neural network, Invasive coronary angiography

## Abstract

**Background:**

Quantitative perfusion cardiovascular magnetic resonance (QP-CMR) allows the generation of pixel-wise myocardial blood flow (MBF) maps using model-based deconvolution with several models including Tofts, modified-Tofts, and Fermi function models. However, the accuracy of pixel-wise MBF mapping has not been fully investigated in humans. The aim of this study was to evaluate the accuracy of advanced QP-CMR using ^15^O-water positron emission tomography (PET) as a reference.

**Methods:**

Thirty-nine patients (29 men, 68 ± 11 years) with known or suspected coronary artery disease underwent both CMR including stress and rest QP-CMR and ^15^O-water PET at a median interval of 13 days. QP-CMR was performed using dual-sequence technique and a single bolus of gadolinium contrast agent during adenosine triphosphate stress and at rest. MBF maps were generated using three different model-based deconvolution techniques as follows: Tofts, modified-Tofts, and Fermi function models. Agreement of MBF and myocardial perfusion reserve (MPR) between QP-CMR and ^15^O-water PET was evaluated using Pearson’s correlation, Bland–Altman analysis, and intraclass correlation (ICC). The ability of CMR-derived stress MBF and MPR to detect PET-defined abnormal myocardial perfusion (stress MBF ≤2.3 mL/min/g and MPR ≤2.5) was evaluated by receiver operating characteristic (ROC) analysis.

**Results:**

CMR-derived MBF showed a good linear correlation with ^15^O-water PET-derived MBF in each of the Tofts, modified-Tofts, and Fermi function models (r = 0.776, 0.752, 0.784, respectively; p<0.001 each) at the patient level. Bland–Altman analysis demonstrated measurement biases for MBF between CMR and ^15^O-water PET of 0.31 ± 0.70, 0.05 ± 0.63, and 0.26 ± 0.68 mL/min/g for the Tofts, modified-Tofts, and Fermi function models, respectively. ICCs were 0.734, 0.747, and 0.750, respectively. The area under the ROC curves for stress MBF derived from the Tofts and Fermi function models (0.921 and 0.914, respectively) was significantly higher than that derived from the modified-Tofts model (0.861; p = 0.003 for both). However, there was no significant difference between the Tofts and Fermi function models (p = 0.618).

**Conclusion:**

Advanced QP-CMR using three different model-based deconvolution techniques demonstrated strong agreement with ^15^O-water PET. Of these techniques, the Fermi function and Tofts models were more effective in detecting abnormal myocardial perfusion as determined by ^15^O-water PET. Considering our results, the model complexity, and its technical availability, the Fermi function model may possess a practical advantage.

## Graphical abstract

**Figure:**
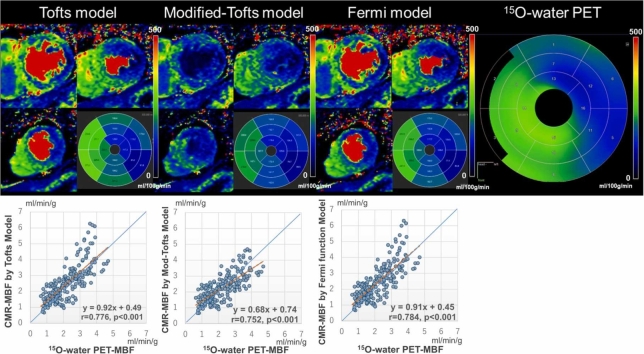

## 1. Introduction

Cardiovascular magnetic resonance imaging (CMR) allows for the noninvasive assessment of myocardial perfusion in patients with suspected coronary artery disease (CAD). The utilization of CMR for this purpose is recommended by current clinical guidelines [1], [2]. In daily clinical practice, CMR perfusion images are typically interpreted through visual assessment. However, quantification of absolute myocardial blood flow (MBF) at rest and during pharmacological stress, as well as the derived myocardial perfusion reserve (MPR) from CMR, has attracted growing interest in recent years [3]. Quantitative perfusion (QP)-CMR analysis offers several advantages over conventional visual interpretation. For one, this method reduces interobserver variability by minimizing dependence on reader expertise and enhances diagnostic confidence [4]. Moreover, this approach enables the detection of subtle regional ischemia that may be overlooked by visual assessment and helps to identify patients with globally reduced perfusion caused by multi-vessel pathology that is often missed in qualitative evaluations [5]. Several studies have emphasized the incremental diagnostic and prognostic value of quantitative CMR perfusion imaging for improving the detection and management of CAD [6], [7]. The utility of QP-CMR for the assessment of coronary microvascular dysfunction (CMD) has also been reported [8], [9]. The recent development of dual-sequence T1 saturation-correction techniques, combined with automated post-processing pipelines, has allowed for pixel-wise MBF mapping based on model-based deconvolution, which is built on the tracer-kinetic theory of linear time-invariant systems [10]. This approach can utilize several different perfusion models, including Tofts, modified-Tofts, and Fermi function models [11], [12]. Recently, the Society for Cardiovascular Magnetic Resonance (SCMR) Expert Consensus Statement on QP-CMR has been published, providing recommendations for the acquisition and analysis of QP-CMR to facilitate standardization of methodology [13]. However, the accuracy of these pixel-wise QP-CMR techniques remains insufficiently validated in human studies and there is currently no clear consensus on the best model for perfusion quantification [13].

Positron emission tomography (PET), particularly using ^15^O-labeled water (^15^O-water), provides a well-established reference standard for absolute MBF quantification. As ^15^O-water is metabolically inert and freely diffusible with a nearly 100% extraction fraction across a wide range of flow rates, this tracer allows most accurate assessment of MBF [14]. An international multicenter study reported that ^15^O-water PET offered high diagnostic performance for detecting significant CAD [15], [16], [17]. In a previous study by Everaars et al. [18], quantitative perfusion CMR using the Fermi function model was compared with ^15^O-water PET. However, this study employed segment-based MBF calculation, not pixel-wise mapping. Consequently, the CMR-derived measurements of stress MBF and MFR showed only modest agreement with those obtained using ^15^O-water PET. Therefore, the accuracy and clinical validity of pixel-wise QP-CMR techniques, including the Fermi function model and other models, against ^15^O-water PET still need to be demonstrated.

The aim of this study was to evaluate the accuracy of QP-CMR-derived pixel-wise MBF mapping using ^15^O-water PET as the reference standard and to compare the diagnostic performance of three different perfusion models in detecting abnormal perfusion defined on ^15^O-water PET.

## 2. Materials and methods

### 2.1. Patient population

This prospective, single-center study was approved by the review board at our institution (approval no. H2021–128), and written informed consent for participation in the study was obtained from all participants prior to enrollment. Sample size was determined based on the intraclass correlation coefficient (ICCs) of MBF as measured by both ^15^O-water PET and CMR in a previous study [18]. The lower bound of the 95% confidence interval (CI) for the ICC of stress MBF obtained by ^15^O-water PET and CMR was 0.17 [16]. The null hypothesis was therefore set as ICC = 0.20. Given the expected similarity or superiority of the ICC in the present study, we set the anticipated ICC at 0.60, referencing an upper bound of 0.59 for the 95%CI reported in the previous study [16]. Assuming a two-sided significance level of α = 0.05 and a statistical power of 0.80, the sample size required to detect a significant ICC (testing the null hypothesis: ICC = 0.20 vs. alternative hypothesis: ICC ‡ 0.20) was calculated to be 35. Allowing for approximately 10% of participants yielding non-evaluable data, the final target sample size was set at 39 subjects.

The following inclusion criteria were: a) participants with suspected or confirmed CAD; and b) age >20 years. The following exclusion criteria were: a) participants for whom iodinated contrast agents or adenosine triphosphate (ATP) were contraindicated; b) participants with persistent arrhythmia; c) participants with acute chest pain or unstable general condition; d) participants with metallic devices; e) participants with a history of cardiovascular events, revascularization procedures, changes to or addition of internal medications between CMR and PET; f) participants with poor image quality from CMR or PET; or g) participants with insufficient pharmacological stress.

**Fig. 1:**
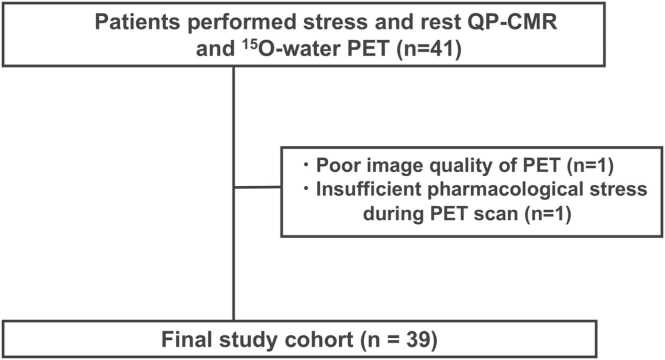
Overview of the study design and patient selection protocol. *QP-CMR* quantitative perfusion cardiovascular magnetic resonance, *PET* positron emission tomography

Forty-one patients with known or suspected CAD underwent both CMR, including stress and rest perfusion and ^15^O-water PET between October 2021 and September 2023. QP-CMR was performed before ^15^O-water PET in all participants. After excluding 1 participant with poor image quality from PET and 1 participant with insufficient pharmacological stress during PET, the final study cohort comprised 39 patients (29 men; mean age, 68 ± 11 years) (Fig. 1). The median interval between CMR and PET was 13 days (interquartile range [IQR], 6–19 days). All participants refrained from caffeine intake for 24 h before QP-CMR and ^15^O-water PET. Heart rate (HR), electrocardiogram (ECG), and blood pressure (BP) were monitored during the examinations.

### 2.2. Cardiovascular magnetic resonance imaging

Cardiovascular magnetic resonance (CMR) studies were performed using a 3.0-T MR scanner (Ingenia 3.0T; Philips Medical Systems, Best, The Netherlands) equipped with dS coils for signal reception. Perfusion imaging was performed using an ECG-triggered single-shot saturation recovery spoiled gradient echo dual-sequence implementation with three high-resolution images in three short-axis slices and an additional interleaved low-resolution image at a basal slice was acquired at every R-R interval over a period of 60 s [11]. Interleaved low-resolution images were acquired between the saturation pulse of the first high-resolution image and image readout, sharing the same saturation pulse between low- and high-resolution images. Low-resolution images were obtained using a fast readout with short saturation recovery time and typical parameters of the following: repetition time (TR), 2.2 ms; echo time (TE), 1.5 ms; saturation recovery time, 25 ms; flip angle, 20°; pixel bandwidth, 1073 Hz; field of view, 340 × 312 mm^2^; acquisition resolution, 1.52 × 6.23 mm^2^; slice thickness, 10 mm; and sensitivity encoding (SENSE) factor, 2.8. High-resolution images were acquired with parameters as reported above, except saturation recovery time was 110 ms and acquisition resolution was 1.52 × 2.55 mm^2^. Both low- and high-resolution data were reconstructed to an in-plane resolution of 1.18 × 1.18 mm^2^. Stress perfusion CMR image acquisition was initiated 5 min after starting ATP infusion (0.16 g/kg/min). Gadoterate meglumine (Gd-DOTA; Guerbet Japan, Tokyo, Japan) was injected at a dose of 0.05 mmol/kg and a flow rate of 4 mL/s, followed by a 20-mL saline flush for stress perfusion CMR. Rest perfusion CMR was performed with an identical set-up at 10 min following stress perfusion CMR. Symptoms, BP, HR, and ECG were monitored during stress scanning. T1 mapping was performed using an steady state free precession (SSFP) single breath hold using a modified look-locker inversion recovery 5 s (3 s) 3 s acquisition scheme at the mid-slice to obtain baseline T1 values before every QP-CMR scan (TR, 2.6 ms; TE, 1.1 ms; flip angle, 35°; field of view, 300 × 330 mm; acquisition matrix, 176 × 141; reconstruction matrix, 352 × 352; SENSE factor, 2; slice thickness, 10 mm) [19]. Late gadolinium enhancement (LGE)-CMR images were acquired 5–10 min after intravenous administration of Gd-DOTA, with a cumulative dose of 0.15 mmol/kg. LGE-MRI were obtained in the LV short-axis planes using an inversion recovery three-dimensional (3D) gradient echo sequence around 5–10 min after the intravenous administration of Gd-DOTA at a cumulative dose of 0.15 mmol/kg [19]. Hematocrit on the day of the CMR was recorded.

### 2.3. MBF quantification by QP-CMR

Dynamic perfusion images first underwent motion correction using the Fast Elastic Image Registration technique [20] and were corrected for coil sensitivity using the acquired proton density maps. Signal intensity (SI) was then converted to gadolinium concentration to account for the SI differences between the low- and high-resolution slices and to correct for nonlinearity between SI and gadolinium concentration [11]. One circular region of interest (ROI) with a 10-mm diameter was manually placed over the motion-corrected low-resolution image with no scaling factor to derive an arterial input function (AIF) curve. The AIF curve was then used to quantify absolute pixel-wise MBF using an automated pipeline employing model-based deconvolution. Three different impulse response functions (IRFs) were used for the model-based deconvolution: the Fermi function model; the Tofts model; and the modified-Tofts model, where perfusion rate (F) or compound transfer constant (K_trans_) represents the magnitude of the function (Table 1) [12]. These metrics (F and K_trans_) were ultimately reported as MBF in the corresponding applications of each model.

**Table 1:** Impulse response function of the three different models

Model	Impulse response function (IRF)
Tofts model	$\mathrm{IRF}\left(t\right)=K_{\mathrm{trans}}\exp^{-(K_{\mathrm{trans}}/V_{e})(t)}$
Modified-Tofts model	$\mathrm{IRF}\left(t\right)=K_{\mathrm{trans}}\exp^{-(K_{\mathrm{trans}}/V_{e})(t)}+V_{p}\partial (t)$
Fermi function model	$\mathrm{IRF}\left(t\right)=\frac{F}{\exp^{k(t-\mathrm{MTT})}+1}$

*IRF* impulse response function, *K_trans_* compound transfer constant, *Ve* extracellular extravascular space volume fraction, *Vp* intravascular plasma volume fraction, F perfusion rate, *k* venous clearance rate for intravascular contrast agent, *MTT* capillary mean transit time

Further CMR image analyses were conducted using CVI42 CMR analysis software (Circle Cardiovascular Imaging, Calgary, Alberta, Canada). Stress and resting MBF maps were manually segmented by a board-certified radiologist (M.T., with 9 years of experience in CMR) blinded to clinical information and results of other diagnostic tests, taking special care to exclude blood pool and extracardiac tissue. MBF and MPR were determined in 16 standard American Heart Association (AHA) segments. The MBF values at segment and vessel levels were calculated as the average MBF for all pixels within each AHA segment and the coronary vessel territory, respectively [21]. MPR was calculated as the stress MBF divided by the resting MBF in each segment. Segments with poor registration and thinned myocardial segments less than 5 mm due to infarction were excluded from the analysis.

### 2.4. ^15^O-water positron emission tomography

The ^15^O-water cardiac PET was performed at rest and during vasodilator stress using a PET/CT scanner (Discovery PET/CT 690 VCT, GE Medical Systems, Milwaukee, Wisconsin) [22]. Through an antecubital intravenous line, 250 MBq of ^15^O-water (7 mL) was administered at an infusion rate of 0.4 mL/s, followed by a 20-mL saline flush at 0.4 mL/s. Dynamic 3D non-gated PET of the heart was performed for 7 min, starting simultaneously with the injection of ^15^O-water. Pharmacological vasodilator stress was performed with intravenous infusion of ATP (0.16 g/kg/min for 10 min). Low-dose slow helical CT (120 kVp, 10 mA, and rotation time of 1.0 s) with breath holding at shallow expiration was performed for attenuation correction. Manual registration was performed by moving PET images over the CT images using dedicated software (ACQC; GE Medical Systems). Dynamic cardiac PET images (28 frames: 18 × 5, 3 × 10, 3 × 20, and 4 × 30 s) were then reconstructed with 3D ordered subsets, expectation maximization (3 iterations, 16 subsets), and a 5-mm Gaussian filter [22]. Reconstructed resolution was 1.95 × 1.95 × 3.34 mm. PET images were analyzed to estimate MBF using Carimas TM analytical software (version 2.8; Turku PET Centre, Turku, Finland) [23] by a board-certified radiologist (Y.I., with 25 years of experience in nuclear cardiac imaging) blinded to clinical information and the results of other diagnostic tests. The MBF values at segment and vessel levels were calculated as the average MBF for all voxels within each segment and the coronary vessel territory based on the 16 AHA model, respectively [21].

### 2.5. Statistical analysis

The normality of continuous variables was assessed using the Shapiro–Wilk test. As all continuous variables showed normal distributions, data for continuous variables are presented as mean ± standard deviation (SD). Categorical variables are presented as frequencies and percentages. The relationship of MBF values between CMR and PET was assessed using the Pearson correlation coefficient and linear regression analysis. Agreement of MBF between CMR and PET was evaluated using the Bland–Altman method, ICC, and equivalence testing. The interpretation of ICC values followed the guidelines in which ICC<0.50 was considered poor, 0.50–0.75 moderate, 0.75–0.90 good, and >0.90 excellent reliability [24]. The equivalence test was performed using two one-sided t-tests [25]. These two one-sided t-tests were constructed for the null hypotheses that the true difference would exceed the equivalent criteria. If both tests rejected the null hypothesis (i.e., p<0.05), the groups would be considered practically equivalent. The greatest of these p-values was taken to evaluate equivalence. The equivalence criteria were: a) within 0.90 mL/min/g for MBF and 0.98 for MPR at the participant level; and b) within 1.43 for stress MBF and 2.80 for MPR at the vessel and segment levels according to the range of variability of MBF measurements in a previous PET study [26].

The diagnostic performance of three different deconvolution methods for detecting abnormal perfusion defined as an MBF ≤2.3 mL/min/g and an MPR ≤2.5 on ^15^O-water PET [15] was evaluated using the area under the receiver operating characteristic (ROC) curve (AUC). The DeLong test was used to compare AUCs. Equivalence tests were performed using R software (version 4.5.0). All other statistical analyses were performed using MedCalc version 20.014 software (MedCalc Software, Ostend, Belgium). Values of p<0.05 were considered to indicate statistical significance.

## 3. Results

### 3.1. Participant characteristics

The background characteristics of patients are shown in Table 2. HR increased with ATP administration from 65 ± 9 beats/min to 78 ± 10 beats/min with QP-CMR (p<0.001), and from 66 ± 10 beats/min to 77 ± 13 beats/min with ^15^O-water PET (p<0.001).

**Table 2:** Patient background.

Patient characteristics		
	Age, years, mean±SD	67.6 ± 11.5
	Male, n(%)	29 (74)
	BMI, kg/m^2^, mean±SD	23.9 ± 4.0
Risk factor of CAD, n(%)		
	Hypertension	32 (82)
	Diabetes	18 (46)
	Hyperlipidemia	22 (56)
	Smoking	26 (65)
	Family history of CAD	8 (21)
Histoy of CAD, n(%)		
	History of PCI	20 (51)
	History of CABG	1 (3)
Medication, n(%)		
	ACE-I	6 (15)
	ARB	18 (46)
	β blocker	15 (38)
	CCB	17 (44)
	Diuretic	8 (21)
	Statin	21 (54)
	Antiplatelet	24 (62)

*SD* standard deviation, *BMI* body mass index, *CAD* coronary artery disease, *PCI* percutaneous coronary intervention, *CABG* coronary artery bypass grafting, *ACE-I* angiotensin-converting enzyme inhibitor, *ARB* angiotensin receptor blocker, *CCB* calcium channel blocker. Data are numbers (%) of cases or means ± standard deviation.

### 3.2. Myocardial blood flow

About 55 of the 624 segments (8.8%) were excluded, comprising 36 segments with poor registration of QP-CMR (5.8%) and 19 segments with a thickness less than 5 mm due to infarction (3.0%).

PET-derived and CMR-derived MBF during stress and rest at participant, vessel, and segment levels were summarized in Table 3. Using criteria based on ^15^O-water PET interstudy variability, CMR-derived sMBF using three different models demonstrated equivalence to PET-derived MBF at each of the participant, vessel, and segment levels (p<0.001 each).

**Table 3:** Perfusion parameters derived from QP-CMR and ^15^O-water PET

	QP-CMR			^15^O-water PET
	Tofts	Modified-Tofts	Fermi function	
Stress MBF, mean±SD, mL/min/g				
Participant level	3.18±0.98	2.73±0.70	3.12±0.97	2.83±0.73
Vessel level	3.18±1.03	2.74±0.76	3.12±1.03	2.83±0.77
Segment level	3.15±1.22	2.70±0.86	3.08±1.20	2.79±0.85
Rest MBF, mean±SD, mL/min/g				
Participant level	1.74±0.56	1.67±0.52	1.71±0.55	1.46±0.45
Vessel level	1.73±0.58	1.66±0.53	1.70±0.57	1.46±0.48
Segment level	1.70±0.63	1.63±0.58	1.67±0.62	1.45±0.52
MPR, mean±SD				
Participant level	1.96±0.66	1.76±0.53	1.95±0.64	2.10±0.68
Vessel level	1.97±0.68	1.77±0.57	1.95±0.66	2.10±0.74
Segment level	1.98±0.78	1.78±0.64	1.96±0.76	2.08±0.80

*QP-CMR* quantitative perfusion cardiac magnetic resonance, *SD* standard deviation, *MBF* myocardial blood flow, *MPR* myocardial perfusion reserve. Data are means ± standard deviation.

**Fig. 2:**
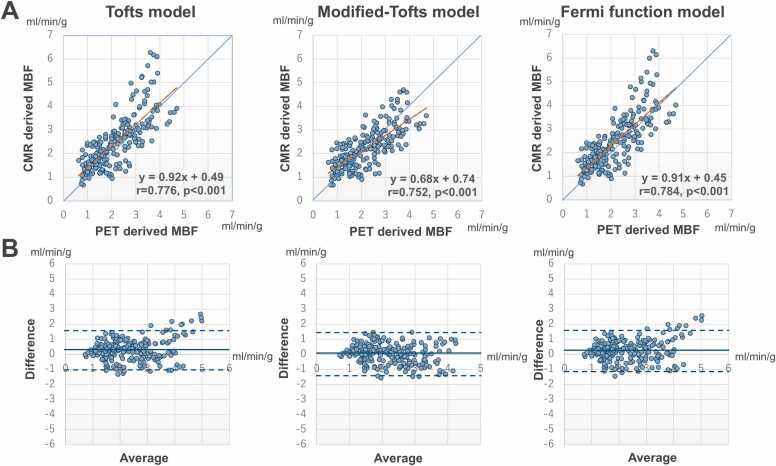
Correlations and agreements of MBF with PET and CMR. **(A)** Correlations of MBF with PET and CMR using Tofts, modified-Tofts, and Fermi function models at the vessel level. The blue line represents the reference line, and the orange line represents the fitted regression line. **(B)** Bland–Altman plots with 95% limits of agreement demonstrate agreement of MBF with PET and CMR at the vessel level. The solid line represents the bias, and the dotted lines represent the 95% limits of agreement. *CMR* cardiovascular magnetic resonance, *PET* positron emission tomography, *MBF* myocardial blood flow

**Fig. 3:**
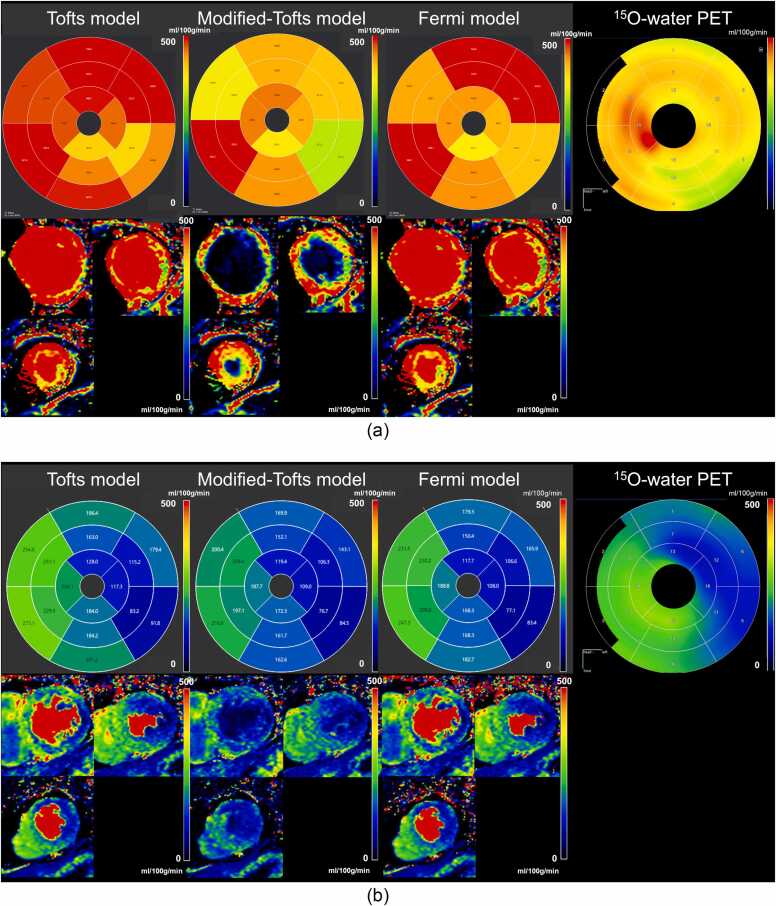
Representative case of stress MBF maps by PET and CMR. A) A 33-year-old man presented with chest discomfort. Three CMR-derived MBF maps and a ^15^O-water PET-derived MBF map demonstrate no abnormal perfusion. B) A 55-year-old man with chronic total occlusions of the diagonal branch and obtuse marginal branches. Three CMR-derived MBF maps and a ^15^O-water PET-derived MBF map demonstrate a similar distribution of abnormal perfusion in the anterolateral to lateral wall corresponding to the diagonal branch and obtuse marginal branch occlusions. *CMR* cardiovascular magnetic resonance, *PET* positron emission tomography, *MBF* myocardial blood flow

A strong linear correlation was seen for MBF between CMR and PET at the participant level (Tofts: y = 0.93x + 0.47; r =0.792, p<0.001; modified-Tofts: y = 0.69x + 0.73; r =0.773, p<0.001; Fermi: y = 0.92x + 0.44; r = 0.799, p<0.001), vessel level (Tofts: y = 0.92x + 0.49; r = 0.776, p<0.001; modified-Tofts: y = 0.68x + 0.74; r = 0.752, p<0.001; Fermi: y = 0.91x + 0.45; r = 0.784, p<0.001), and segment level (Tofts: y = 0.84x + 0.65; r =0.673, p<0.001; modified-Tofts: y = 0.62x + 0.85; r = 0.667, p<0.001; Fermi: y = 0.83x + 0.62; r =0.679, p<0.001) (Fig. 2A). Bland–Altman analysis demonstrated that the measurement bias of MBF between CMR and PET was 0.31 ± 0.66 mL/min/g, 0.06 ± 0.59 mL/min/g, and 0.27 ± 0.64 mL/min/g at the participant level; 0.31 ± 0.70 mL/min/g, 0.05 ± 0.63 mL/min/g, and 0.26 ± 0.68 mL/min/g at the vessel level; and 0.31 ± 0.91 mL/min/g, 0.05 ± 0.77 mL/min/g, and 0.26 ± 0.89 mL/min/g at the segment level for the Tofts, modified-Tofts, and Fermi models, respectively (Fig. 2B). ICCs for MBF measurements between CMR and PET were 0.747 (95%CI, 0.567–0.848), 0.768 (95%CI, 0.659–0.846), and 0.764 (95%CI, 0.615–0.854) at the participant level, 0.734 (95%CI, 0.601–0.816), 0.747 (95%CI, 0.685–0.799), and 0.750 (95%CI, 0.644–0.821) at the vessel level, and 0.633 (95%CI, 0.549–0.698), 0.665 (95%CI, 0.631–0.696), and 0.647 (95%CI, 0.582–0.701) at the segment level for the Tofts, modified-Tofts, and Fermi models, respectively. A representative participant is presented in Fig. 3.

### 3.3. Myocardial perfusion reserve

PET-derived and CMR-derived MPRs at participant, vessel, and segment levels were summarized in Table 3. Using criteria based on ^15^O-water PET interstudy variability, the CMR-derived MPR obtained from the three different models demonstrated equivalence to PET-derived MPR at the participant, vessel, and segment levels (p<0.001 each).

**Fig. 4:**
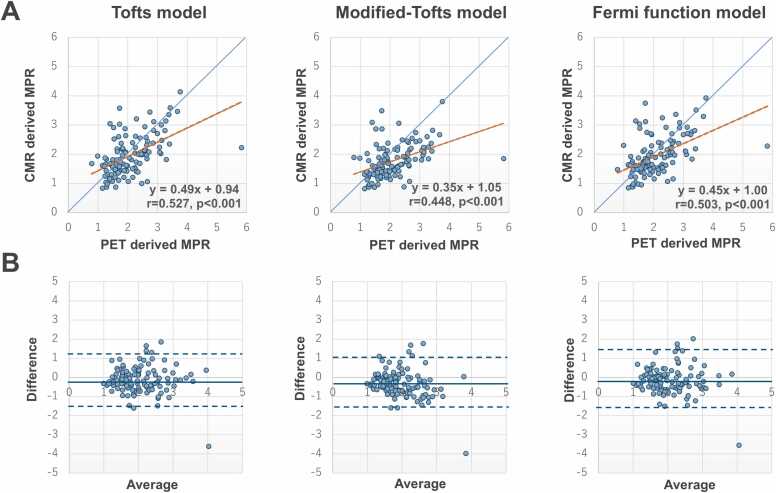
Correlations and agreements of MPR with PET and CMR. **(A)** Correlations of MPR with PET and CMR using Tofts, modified-Tofts, and Fermi function models at the vessel level. The blue line represents the reference line, and the orange line represents the fitted regression line. **(B)** Bland–Altman plots with 95% limits of agreement demonstrate agreement of MPR with PET and CMR at the vessel level. The solid line represents the bias, and the dotted lines represent the 95% limits of agreement. *CMR* cardiovascular magnetic resonance, *PET* positron emission tomography, *MPR* myocardial perfusion reserve

A moderate linear correlation of MPR was seen between CMR and PET at the participant level (Tofts: y = 0.55x + 0.81, r = 0.563, p<0.001; modified-Tofts: y = 0.38x + 0.96, r = 0.486, p = 0.002; Fermi function: y = 0.51x + 0.89, r = 0.535, p<0.001), vessel level (Tofts: y = 0.49x + 0.94, r = 0.527, p<0.001; modified-Tofts: y = 0.35x + 1.05, r = 0.448, p<0.001; Fermi function: y = 0.45x + 1.00, r = 0.503, p<0.001), and segment level (Tofts: y = 0.46x + 1.01, r = 0.474, p<0.001; modified-Tofts: y = 0.33x + 1.10, r = 0.406, p<0.001; Fermi function: y = 0.43x + 1.08, r = 0.446, p<0.001) (Fig. 4A). Bland–Altman analysis demonstrated that the measurement bias of MPR between CMR and PET was −0.14 ± 0.62, −0.34 ± 0.63, and −0.15 ± 0.64 at the participant level, −0.13 ± 0.69, −0.32 ± 0.70, and −0.14 ± 0.70 at the vessel level, and −0.10 ± 0.81, −0.30 ± 0.80, and −0.12 ± 0.82 at the segment level for the Tofts, modified-Tofts, and Fermi function models, respectively (Fig. 4B). ICCs for MPR measurements between CMR and PET were 0.557 (95%CI, 0.302–0.739), 0.415 (95%CI, 0.108–0.648), and 0.527 (95%CI, 0.263–0.719) at the participant level, 0.519 (95%CI, 0.373–0.640), 0.389 (95%CI, 0.191–0.549), and 0.492 (95%CI, 0.342–0.618) at the vessel level, and 0.470 (95%CI, 0.403–0.532), 0.365 (95%CI, 0.253–0.461), and 0.441 (95%CI, 0.371–0.505) at the segment level for the Tofts, modified-Tofts, and Fermi function models, respectively.

### 3.4. Diagnostic performance of abnormal perfusion

**Fig. 5:**
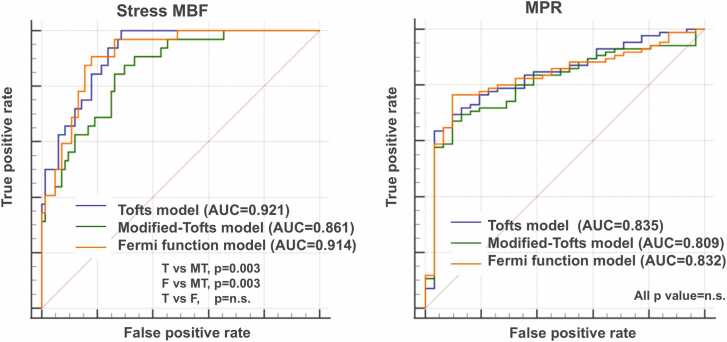
ROC curves of stress MBF and MPR for detecting abnormal myocardial perfusion, defined as ^15^O-water PET-derived stress MBF ≤ 2.3 mL/min/g and MPR ≤ 2.5. *MBF* myocardial blood flow, *MPR* myocardial perfusion reserve, *ROC* receiver operating characteristic

CMR-derived stress MBF and MPR at the vessel level demonstrated AUCs of 0.921 and 0.835 for the Tofts model, 0.861 and 0.809 for the modified-Tofts model, and 0.914 and 0.832 for the Fermi function model, respectively, for the detection of abnormal myocardial perfusion as determined by ^15^O-water PET (Fig. 5). The AUCs of stress MBF derived from the Tofts model and Fermi function model were significantly higher than those derived from the modified-Tofts model (p = 0.003 each), whereas no significant difference was observed between the Tofts and Fermi function models (p = 0.618). In addition, the AUCs of MPR did not differ significantly among the three models (p = n.s.).

## 4. Discussion

The main findings of this study were as follows. First, CMR-derived MBF showed good agreement with ^15^O-water PET-derived MBF in each of the Tofts, modified-Tofts, and Fermi function model-based deconvolution techniques. Second, CMR-derived MBF and MPR using Tofts, modified-Tofts, and Fermi function models accurately detected abnormal myocardial perfusion as determined by ^15^O-water PET. Tofts and Fermi function models showed higher AUCs compared to the modified-Tofts model in detecting abnormal myocardial perfusion.

The recent development of dual-sequence T1 saturation-correction techniques with automated post-processing pipelines has enabled the generation of pixel-wise MBF maps, thereby accelerating the clinical adoption of QP-CMR. However, despite the growing clinical use, only a single study has rigorously validated the accuracy of pixel-wise MBF mapping. Engblom et al. evaluated QP-CMR-derived MBF using deconvolution with a blood tissue exchange model, making comparisons with ^13^N-NH_3_ PET in 21 patients with stable CAD. The results showed good agreement of MBF values between CMR and PET (−0.1 ± 0.5 mL/min/g at the participant level and −0.1 ± 0.6 mL/min/g at the segment level), with strong correlations observed at both levels (participant level: r = 0.92, p<0.001; segment level: r = 0.83, p<0.001). Further, significant correlations were found for MPRs between the two modalities (participant level: r = 0.69, p = 0.001; segment level: r = 0.57, p<0.001, respectively) [27]. In our study, pixel-wise MBF mapping using the dual-sequence technique and three different perfusion models, conducted under a different MR setting, similarly demonstrated good agreement with ^15^O-water PET. The correlations observed for MBF (r = 0.752 to 0.784) and MPR (r = 0.448 to 0.503) at the vessel level were consistent with those obtained by Engblom et al. using a blood tissue exchange model and a different PET tracer (^13^N-NH^3^), further supporting the validity of pixel-wise MBF mapping using QP-CMR. These findings contribute important additional evidence toward the clinical reliability of this technique across different imaging platforms.

Further, we demonstrated that CMR-derived MBF provided good agreement with ^15^O-water PET-derived MBF across the Tofts, modified-Tofts, and Fermi function models. The Fermi function model is a widely used approach for QP-CMR [4], [5], [7], [9], [27]. The model is derived from indicator dilution theory and models the IRF using the Fermi function, which captures the characteristic first-pass dynamics of an intravascular tracer. This model estimates key physiological parameters such as MBF and mean transit time without explicitly defining distinct tissue compartments. In contrast, the Tofts and modified-Tofts models are compartment-based and describe the kinetics of contrast agent exchange between plasma and the extravascular extracellular space assuming diffusion-limited exchange. The standard Tofts model includes two parameters, transfer constant (K_trans_) and extracellular volume fraction (Ve), assuming a negligible plasma volume. The modified-Tofts model incorporates the plasma volume fraction (Vp) for greater accuracy in highly vascular tissues. These models were initially developed to investigate brain and brain tumor perfusion. Since these models do not explicitly model flow-limited kinetics or capillary transit times, they are less physiologically appropriate for myocardial perfusion analysis in theory. However, in our study, the Tofts model and Fermi function model produced comparable MBF values, whereas the modified-Tofts model yielded lower MBF estimates compared to the Tofts and Fermi function models. This finding is consistent with previous studies. Schwab et al. [28] investigated patients with suspected CAD and found that MBF values derived from the Tofts model closely matched those obtained from the Fermi function model, attributing this similarity to the fact that, when delay time is properly taken into account, the Fermi function model essentially describes a 1-compartment situation like the Tofts model. Thus, despite its limited physiological assumptions, the Tofts model has been used in cardiac perfusion CMR and has shown acceptable performance. It is also important to note that the Fermi function model, while widely used, is a purely mathematical function that likewise does not explicitly represent myocardial perfusion physiology. In our study, the modified-Tofts model yielded lower MBF estimates compared to the Tofts and Fermi function models in our study. Pack et al. [29] reported that MBF values obtained using the Fermi function model were higher than those derived from the modified-Tofts model. They explained that the Fermi model produced perfusion estimates approximately 25% higher when all measured data were used in the analysis, compared to when only the first pass of dynamic enhancement data was used, because the Fermi function model does not properly account for delayed contrast efflux from extravascular tissue. To the best of our knowledge, this is the first study to validate pixel-wise MBF mapping using the Tofts and modified-Tofts pharmacokinetic models. A key strength and novelty of our work lies in the direct comparison of these models with the established Fermi function model, using ^15^O-water PET as a reference within the same patient population. By demonstrating the feasibility of alternative perfusion models for pixel-wise MBF mapping, our findings may help broaden the methodological basis for QP-CMR and support further standardization efforts.

Quantitative MBF measurement using ^15^O-water PET has been shown to provide high diagnostic performance in detecting hemodynamically significant CAD [15], [16], [17]. The PACIFIC 2 study, a prospective, head-to-head comparison of qualitative stress perfusion CMR, qualitative ^99^ᵐTc-SPECT, and quantitative ^15^O-water PET using fractional flow reserve (FFR) < 0.8 as the reference standard, demonstrated the superiority of quantitative PET in terms of AUC and diagnostic accuracy (AUC: 0.76, 0.66, and 0.66; accuracy: 70%, 70%, and 67%, respectively) [17]. One of the key advantages of PET in this setting was its ability to provide quantitative assessment, which likely contributed to its superior diagnostic performance. In our study, we demonstrated that CMR-derived MBF and MPR, quantified using Tofts, modified-Tofts, and Fermi function models, could accurately detect abnormal myocardial perfusion as defined by ^15^O-water PET. These findings suggest that when quantified, CMR has the potential to achieve diagnostic performance comparable to that of PET for identifying significant obstructive CAD. The in-plane reconstructed spatial resolution of perfusion CMR is higher than that of ^15^O-water PET, which may enable improved detection of subendocardial ischemia. However, perfusion CMR was acquired with only three short-axis slices (base, mid, and apex), whereas PET provides fully volumetric 3D MBF. Therefore, PET may offer a more accurate assessment of the overall extent of ischemic burden. Previous papers showed that the diagnostic performance in the detection of significant CAD was comparable between QP-CMR and ^15^O-water PET [15], [30], [31]. Future studies directly comparing the diagnostic performance of QP-CMR and PET are warranted. Among the three kinetic models evaluated, Tofts and Fermi function models demonstrated higher AUCs compared to the modified-Tofts model in detecting abnormal perfusion. A possible explanation for this discrepancy lies in the complexity of the model. The modified-Tofts model includes a greater number of parameters to allow direct physiological interpretation of permeability and tissue volumes. However, this added complexity may result in parameter constraints during fitting, potentially limiting diagnostic performance in clinical settings. The recently published SCMR Expert Consensus Statement on QP-CMR indicates that perfusion models with fewer fitting parameters generally tend to be more robust [13]. Our results demonstrated that the Tofts and Fermi function models achieved higher performance than the modified-Tofts model, which has more fitting parameters, in detecting abnormal myocardial perfusion, as determined by ^15^O-water PET. Conversely, the accuracy and precision in calculating the MBF and MPR were otherwise comparable among the three models. Among the three models evaluated, the Fermi function model has been most widely adopted in previous studies [4], [11], [12], [18], [28], [29], [30] and is already implemented in several research software platforms. This broad implementation may facilitate wider clinical deployment. Therefore, the Fermi function model may possess a practical advantage. Nevertheless, further validation is needed to assess the suitability of perfusion modeling for the diagnosis of obstructive CAD.

In recent years, interest has been growing in the evaluation of CMD, reflecting its increasingly recognized role in patients presenting with chest pain but without obstructive CAD. Stress perfusion CMR provides valuable insights into the physiological status of both the epicardial coronary arteries and the coronary microcirculation [3]. In particular, the presence of stress-induced perfusion abnormalities in the absence of epicardial CAD may indicate underlying CMD. QP-CMR has emerged as a promising noninvasive tool for the assessment of CMD. Rahman et al. [8] investigated 75 patients with angina and no obstructive CAD, using invasive coronary flow reserve (CFR) <2.5 as the diagnostic standard for CMD. Their findings demonstrated that MPR derived from QP-CMR showed high diagnostic accuracy and outperformed qualitative visual assessment. Further evidence from Kotecha et al. [9] supported the utility of QP-CMR in distinguishing CAD from CMD. In that study, stress MBF ≤1.94 mL/min/g was predictive of significant CAD, while a global stress MBF <1.82 mL/min/g was helpful in identifying CMD. Notably, these studies primarily employed the Fermi function model for perfusion quantification. To date, evaluations using alternative kinetic models such as the Tofts model or modified-Tofts model have been limited. Given the potential impact of model selection on perfusion quantification and diagnostic performance, further validation studies are warranted to assess the suitability and accuracy of various perfusion models, particularly in the diagnosis of CMD.

## 5. Limitations

Several limitations of the present study must be kept in mind. First, we did not assess the diagnostic utility of the three different deconvolution methods in the clinical management of patients with CAD and CMD. Further investigations are warranted to evaluate their impact on diagnosis with reference to invasive physiological parameters obtained using catheter-based techniques. Second, MBF values may vary across different systems. Nevertheless, our quantification method is applicable as long as a similar MR scanner, imaging protocol, and contrast medium injection protocol to those used in this study are employed. To the best of our knowledge, most previous QP-CMR studies have adopted imaging and injection protocols comparable to ours. In this regard, our quantification approach remains compatible with widely used QP-CMR protocols.

## 6. Conclusions

CMR-derived MBF showed good agreement with ^15^O-water PET-derived MBF in each of the Tofts, modified-Tofts, and Fermi function model-based deconvolution techniques. QP-CMR using the Fermi function and Tofts models more accurately detected abnormal myocardial perfusion as determined by ^15^O-water PET. These results in the present study demonstrate that advanced QP-CMR using model-based deconvolutions is an accurate imaging technique for the assessment of myocardial perfusion. Considering our results, the model complexity, and its technical availability, the Fermi function model may possess a practical advantage.

## Funding

This work was supported by JSPS KAKENHI Grant Number 21K07564.

## Author contributions

**Masafumi Takafuji:** Writing – original draft, Visualization, Investigation, Formal analysis, Data curation. **Masaki Ishida:** Writing – review & editing, Methodology, Investigation, Formal analysis, Data curation, Conceptualization. **Yasutaka Ichikawa:** Methodology, Investigation. **Satoshi Nakamura:** Investigation. **Haruno Ito:** Investigation. **Takanori Kokawa:** Investigation. **Suguru Araki:** Investigation. **Shintaro Yamaguchi:** Investigation. **Naoki Hashimoto:** Investigation. **Shiro Nakamori:** Investigation. **Tairo Kurita:** Investigation. **Kaoru Dohi:** Investigation. **Hajime Sakuma:** Writing – review & editing, Supervision, Project administration, Conceptualization.

## Ethics approval and consent

This study was conducted in accordance with the principles of the Declaration of Helsinki and with the approval of the Mie University Institutional Review Board at Mie University Hospital (approval no. H2021–128), and written informed consent for participation in the study was given by all participants.

## Consent for publication

Not applicable.

## Declaration of competing interests

The authors declare the following financial interests/personal relationships which may be considered as potential competing interests: Hajime Sakuma reports financial support was provided by JSPS KAKENHI (Grant Number JP20K12345). If there are other authors, they declare that they have no known competing financial interests or personal relationships that could have appeared to influence the work reported in this paper.

## Data Availability

The datasets used and/or analyzed during the current study are available from the corresponding author on reasonable request.
